# Supplementary material for: A systematic investigation of detectors for low signal-to-noise ratio EMG signals
Source: F1000Res. 2024 Apr 18;12:429. Originally published 2023 Apr 21. [Version 4] doi: 10.12688/f1000research.132382.4 (PMC10997989; doi:10.12688/f1000research.132382.4)
Supplement: Peer review history

# Reviewer report: Reviewer response for version 3

**DOI:** 10.5256/f1000research.164086.r258897

**Published:** 2024-04-05

**Authors:** Werner Wolf

**Affiliations:** 1 Universitat der Bundeswehr Munchen, Neubiberg, Bavaria, Germany

The manuscript is now in shape, even some points (e.g. the cost definition) can be discussed critically.

 One point can be solved easily: it sounds really inconsistant to measure a sEMG at no residual movement. EMG activity shows muscle activity and muscle activity means force at a joint. No residual movement at EMG activity can be caused by

 - resolution of the movement measurement is limited

 - friction of the joint to high or isometric condition

 - cocontraction (which is probably in stroke)

 The authors should mention this point shortly and they should use the term "no visible movement" as they do on page 15 para 4, line 3. Use this formulation always.

 Maybe, references 7, 9 and 33 need formal corrections.

Is the work clearly and accurately presented and does it cite the current literature?

Partly

If applicable, is the statistical analysis and its interpretation appropriate?

Partly

Are all the source data underlying the results available to ensure full reproducibility?

Yes

Is the study design appropriate and is the work technically sound?

Yes

Are the conclusions drawn adequately supported by the results?

Yes

Are sufficient details of methods and analysis provided to allow replication by others?

Yes

Reviewer Expertise:

Biosignal processing, human motor control

I confirm that I have read this submission and believe that I have an appropriate level of expertise to confirm that it is of an acceptable scientific standard.

# Reviewer report: Reviewer response for version 2

**DOI:** 10.5256/f1000research.161421.r242339

**Published:** 2024-02-16

**Authors:** Vincent Crocher

**Affiliations:** 1 The University of Melbourne, Melbourne, Victoria, Australia

I have no more comments.

Is the work clearly and accurately presented and does it cite the current literature?

Yes

If applicable, is the statistical analysis and its interpretation appropriate?

Yes

Are all the source data underlying the results available to ensure full reproducibility?

Yes

Is the study design appropriate and is the work technically sound?

Yes

Are the conclusions drawn adequately supported by the results?

Partly

Are sufficient details of methods and analysis provided to allow replication by others?

Yes

Reviewer Expertise:

Rehabilitation robotics

I confirm that I have read this submission and believe that I have an appropriate level of expertise to confirm that it is of an acceptable scientific standard.

# Reviewer report: Reviewer response for version 1

**DOI:** 10.5256/f1000research.145296.r223113

**Published:** 2024-02-19

**Authors:** Werner Wolf

**Affiliations:** 1 Universitat der Bundeswehr Munchen, Neubiberg, Bavaria, Germany

First of all, I would like to confirm the detailled review of Vincent Crocher. But I would like to underline his criticism on the lengthy elaboration of the rehabilitation issue. The manuscript focusses on event detection in stochastic signals and, therefore, it can be mapped to the biosignal processing field. The EMG processing  is only specificly addressed by the inclusion of the biophysical signal model. And the topic „robots in rehalitation“ is a separate field not really related main issue of this manuscrpt.

 Thus, the total rehabilitation part – not only the BCI passages -  can be reduced – as Vincent already states – to a single sentence and a removal of about 25 references.

 It sounds also contradicting to refer to the case „without residual movement“ but to measure surface EMG. What presumably is addressed by the manuscript are combined muscle excitations; i.e. to generate stiffness in the arm when fingers are grasping an object. But such a stiffness in the forearm is ambiguous, because the same stiffness will be generated with different finger movements. I will not continue this rehabilitation issue, because it is not the concern of this manuscript. – as mentioned above.

 Summarizing: The main results are interesting and they should be provided in a compressed form.

Is the work clearly and accurately presented and does it cite the current literature?

Partly

If applicable, is the statistical analysis and its interpretation appropriate?

Partly

Are all the source data underlying the results available to ensure full reproducibility?

Yes

Is the study design appropriate and is the work technically sound?

Yes

Are the conclusions drawn adequately supported by the results?

Yes

Are sufficient details of methods and analysis provided to allow replication by others?

Yes

Reviewer Expertise:

Biosignal Processing - human motor control

I confirm that I have read this submission and believe that I have an appropriate level of expertise to confirm that it is of an acceptable scientific standard, however I have significant reservations, as outlined above.

# Response

**Published:** 2024-03-20

**Authors:** Monisha Yuvaraj

**Affiliations:** Applied Mechanics, Indian Institute of Technology Madras, Chennai, Tamil Nadu, India

**1. Summary**

 Thank you very much for taking the time to review this manuscript. Please find the detailed responses below and the corresponding revisions/corrections highlighted/in track changes in the re-submitted files.

**2. Point-by-point response to Comments and Suggestions for Authors**

 In the following, the reviewer’s comments are in Italics which is followed by our response.

- *First of all, I would like to confirm the detailled review of Vincent Crocher. But I would like to underline his criticism on the lengthy elaboration of the rehabilitation issue. The manuscript focusses on event detection in stochastic signals and, therefore, it can be mapped to the biosignal processing field. The EMG processing  is only specificly addressed by the inclusion of the biophysical signal model. And the topic „robots in rehalitation“ is a separate field not really related main issue of this manuscrpt. Thus, the total rehabilitation part – not only the BCI passages -  can be reduced – as Vincent already states – to a single sentence and a removal of about 25 references.*

**Response:** Our primary goal is to identify a suitable EMG detector for on-off control of robotic assistance during therapy. This paper focusses on the problem of event detection of EMG signals as this is the first step towards implementing an effective closed loop control of robotic assistance using EMG. The result of this analysis identifies the most promising detectors for application in EMG-driven robot assisted therapy. The “cost” for identifying the detectors is also designed accordingly. Therefore, in the introduction we have given the background about the necessity to identify the best detector that works well with low SNR EMG signals. However, we have reduced the introduction and discussion section which now highlights only the need for identifying the best detector for application such as EMG driven robot assisted therapy.

**Original text**: Substantial recovery of sensory-motor function after a stroke is possible with high-intensity and high-dosage movement training. ^1^ Rehabilitation robots can facilitate such high-intensity movement training while providing physical assistance to the user for completing movements consistently and precisely. About 30% of stroke survivors are severely impaired ^2^^ ,^^3^ and require physical assistance to actively engage in movement training. While physical assistance from a robot can motivate such subjects to attempt and train movements, they can also provide inappropriately timed or too much assistance ^4^^ ,^^5^ leading to slacking, where patients reduce their voluntary effort and exploit robotic assistance to perform the movements. Inappropriately timed robotic assistance also alters the patient’s sense of agency or subjective awareness of control. This could lead to a lack of intrinsic motivation and attention, affecting motor learning and performance. ^6^ Positive therapeutic effects have been observed only when patients actively engage in therapy. ^7^^ –^^10^ Jo *et al.* ^11^ reported no improvement in clinical scales with passive range of motion therapy. Thus, active patient participation during movement therapy is an essential ingredient for sensorimotor recovery. Robotic assistance contingent on a subject’s intention to move is an effective way to guide neuroplasticity.

**Reduced text in the manuscript:**

 Active patient participation during movement therapy is an essential ingredient for sensorimotor recovery after a stroke. About 30% of stroke survivors are severely impaired ^2 ,^ ^3 ^and require physical assistance to actively engage in movement training. Robotic assistance can motivate such subjects to attempt and train movements and when it is contingent on the intention to move it may be an effective way to guide neuroplasticity. ^12^

  

- *It sounds also contradicting to refer to the case „without residual movement“ but to measure surface EMG. What presumably is addressed by the manuscript are combined muscle excitations; i.e. to generate stiffness in the arm when fingers are grasping an object. But such a stiffness in the forearm is ambiguous, because the same stiffness will be generated with different finger movements. I will not continue this rehabilitation issue, because it is not the concern of this manuscript. – as mentioned above.*
- *Summarizing: The main results are interesting, and they should be provided in a compressed form.*

**Response:** The study by Balasubramanian et al [1], about 70% of severely impaired stroke patients without residual movement in fingers and wrist had residual EMG.

 The lack of residual movements despite the presence of EMG in stroke patients could be because of several reasons, one such is co-contraction in which case we could still pick up residual EMG. We agree with the reviewer that the same stiffness can be generated with different finger movements. However, our objective is not to decode stiffness, but to see if there is EMG activity in a muscle of interest – a muscle the patient is trying to activate while attempting a movement.

 The patient could be activating other (antagonists or unrelated) muscles as well, but this is not relevant to the question of whether the muscle of interest displays EMG activity when it is being activated. We are primarily interested in the most appropriate detector for low SNR EMG from any given muscle, irrespective of what happens with other muscles. Once such a detector is identified, different forms of therapeutic interventions become possible, where patients could be asked to attempt activation of certain muscles while suppressing others.

# Reviewer report: Reviewer response for version 1

**DOI:** 10.5256/f1000research.145296.r219586

**Published:** 2023-11-06

**Authors:** Vincent Crocher

**Affiliations:** 1 The University of Melbourne, Melbourne, Victoria, Australia

The authors propose to evaluate and compare 13 sEMG onset detectors specifically on low signal-to-noise EMG (0db and -3db). The authors propose a thorough work and use an appropriate methodology (including generation of simulated EMG signals, wide range of detectors and tuning, outcome evaluation cost and analysis) which is aligned with the objective of the paper and the application (the use of EMG onset for rehabilitation robotics control).

 The paper is clearly of interest in the field of neuro-rehabilitation robotics and I believe that both the results and the methodology used (and associated code provided, which is reasonably documented) are of direct interest to the field.

 Specific comments:

**Introduction:**

- The part on BCI drawbacks can be shorten into a simple sentence to keep the introduction more straightforward (this is not the focus of the paper).
- "In this paper, EMG always refers to surface-recorded muscle": this indeed makes sense for the neuro-rehabilitation application of interest here and even more generally given the wider adoption and ease of use of surface EMG.I would thus suggest to bring this statement as early as in the introduction and adopt the sEMG acronym across the paper as this is an unambiguous terminology widely used.

**Methods:**

- In the "Simulation of surface EMG signal" section, I would suggest to move the part of the last paragraph ("The phenomenological models were based on the work of De Luca...") to the next section to ease the reading. This part is relative to the Phenomenological models only.
- Figure 1 presenting examples of the three types of generated signals show the biophysical signal of a smaller (20 times) than the phenomenological ones. While the absolute amplitude likely does not affect the detectors, this is slightly confusing. Is there any valid reason for this difference or can these three signals be equalised to the same amplitude?
- In Table 1, authors refer to 'Weights' for \alpha whereas it appears that there is only a single value for each detector. Please clarify.
- In Table 1, while RMS and Lidierth detectors do use a filter for the conditioning, those filters cut-off frequencies are not listed as parameters. Do authors intentionally chose fixed, non-optimised, frequencies for those (and if so, why)? or is this a simple oversight in the table?
- After Eq 12, authors should clearly introduce \alpha and refer to it as weight as this is the fist time it is properly introduced. I would also recommend to explicit it earlier on, as the Weight (weights?) are introduced in Table 1 earlier and earlier in the text but without explanation making it hard to follow.

**Methods: On the cost definition:**

- According to Eq. 16, f(deltat) is 0 (ideal) when the detection happens before the actual onset: \deltat<0ms and so is effectively a False positive. While I understand that this cannot be avoided with the chosen structure of the cost: this suggests that the within the overall cost, the latency and the false negative rates are more redundant whereas the false negative rate is quite independent of the other two.
- More generally, as the authors show that most detectors already behave quite poorly for such low SNR sEMG, the chosen cost bounds, and especially the 20% seems quite high for actual use. Given the application in mind where the objective is to detect one true onset as quickly as possible (and 50ms appears reasonable for a robotics assistance), I wonder if a cost accounting for a more strict false negative rate and more tolerant (if accounted at all) for false positive would make more sense. Indeed, only the earliest positive detection after onset can be considered useful for an application using this signal as a trigger. Wouldn't it make sense to also train and evaluate the detectors on a cost based solely on a very strict false positive rate (5%?) and not included FN in the cost (given that only the earliest detection after true onset matters)? Regarding the FP and FN rates, those are also calculated per trial, meaning that 20% (or even 5%) can mean that each trial has at least several FP before onset and not that 20% (or 5%) of the trials do have a FP. This is this latest figure that is of direct interest for the application. Authors should clearly discuss this point on how these results translate to real scenarios and provide results showing in what percentage of test trials FP detection happens before onset.

**Discussion:**

- It could be interesting to discuss the possibility that the signal processing (detectors) might not be necessarily implemented at the same frequency than the one the sEMG data is collected. Indeed it is common in existing systems to have an EMG acquisition faster than the robotic controller (typically a >2kHz EMG sampling for a 1kHz controller). Does authors have clues on how that might differently affect the different detectors?
- While the use of simulated (generated) EMG signals to evaluate the performance of the detector is appropriate and allows for robust and repeatable outcomes, it would be an interesting addition to also perform a short evaluation on actual low signal-to-noise EMG datasets.
  On that point of potential difference with actual EMG signals, authors rightly point that they here test the optimal behaviour of the detectors, i.e. they would likely all perform worst on actual signals, especially when muscle activation is not constant over a long period. Can authors infer and discuss some difference in performance between the detectors for short activation (typically flickers that can be encountered in rehabilitation applications)? Some detectors are based on relatively long windows of 150ms, and while authors explain in the discussion that the best detectors have a latency of <50ms and so likely suitable for short activation bursts, how this would affect the detectors with longer window? Could they be likely be optimised differently for such signals?

**Minor/typos:**

-  "Devasahayam S43"  => "Devasahayam43"
-  In Table 2, some window sizes are in (ms) some in (s)

Is the work clearly and accurately presented and does it cite the current literature?

Yes

If applicable, is the statistical analysis and its interpretation appropriate?

Yes

Are all the source data underlying the results available to ensure full reproducibility?

Yes

Is the study design appropriate and is the work technically sound?

Yes

Are the conclusions drawn adequately supported by the results?

Partly

Are sufficient details of methods and analysis provided to allow replication by others?

Yes

Reviewer Expertise:

Rehabilitation robotics

I confirm that I have read this submission and believe that I have an appropriate level of expertise to confirm that it is of an acceptable scientific standard, however I have significant reservations, as outlined above.

# Response

**Published:** 2024-01-14

**Authors:** Monisha Yuvaraj

**Affiliations:** Applied Mechanics, Indian Institute of Technology Madras, Chennai, Tamil Nadu, India

**1. S** **ummary**

 Thank you very much for taking the time to review this manuscript. Please find the detailed responses below and the corresponding revisions/corrections highlighted/in track changes in the re-submitted files.

**2. Point-by-point response to Comments and Suggestions for Authors**

 In the following, the reviewer’s comments are in Italics. Each comment has the response and The changes in the manuscript are presented by first showing verbatima segment of the text from the previous manuscript  followed by the modified text. Please find the response as a PDF file here.

 The authors propose to evaluate and compare 13 sEMG onset detectors specifically on low signal-to-noise EMG (0db and -3db). The authors propose a thorough work and use an appropriate methodology (including generation of simulated EMG signals, wide range of detectors and tuning, outcome evaluation cost and analysis) which is aligned with the objective of the paper and the application (the use of EMG onset for rehabilitation robotics control). The paper is clearly of interest in the field of neuro-rehabilitation robotics and I believe that both the results and the methodology used (and associated code provided, which is reasonably documented) are of direct interest to the field.

**Introduction:**

- *The part on BCI drawbacks can be shorten into a simple sentence to keep the introduction more straightforward (this is not the focus of the paper).*

**Response: **We have shortened this segment in the introduction section as suggested.

**Original text**:

 They have a poor signal-to-noise ratio (SNR) with large trial-to-trial intra-subject variability. ^20^ The use of sophisticated signal processing techniques can increase the delay in intent detection, which can result in inappropriately timed robotic assistance leading to suboptimal recovery ^12^^ ,^^21^

 EEG-BCI modalities do not precisely identify which limb segment is intended to move due to its low information rate. ^21^

 Event-related desynchronization of the EEG sensorimotor rhythm may not necessarily reflect movement intention. ^22^

 Critical for practical use, current EEG-BCI systems are too complex and time-consuming for clinical work. ^23^

**Change in the manuscript:** They often exhibit a poor signal-to-noise ratio, with significant trial-to-trial intra-subject variability [20]. Unlike EMG systems, EEG-BCI modalities lack task specificity [21]. Moreover, their complexity and time-consuming nature prevent their routine clinical use [23].

- *"In this paper, EMG always refers to surface-recorded muscle": this indeed makes sense for the neuro-rehabilitation application of interest here and even more generally given the wider adoption and ease of use of surface EMG. I would thus suggest to bring this statement as early as in the introduction and adopt the sEMG acronym across the paper as this is an unambiguous terminology widely used.*

**Response:**

 We have moved the sentence to the introduction and used “sEMG” throughout the paper.

**Change in the manuscript:**

 In this paper, we consider surface-recorded muscle electromyogram activity (sEMG), as this modality will most likely be employed in routine robot-assisted therapy. sEMG could be used as a viable alternative to address the drawbacks of EEG-BCI for robot-assisted therapy.

**Methods:**

- *In the "Simulation of surface EMG signal" section, I would suggest to move the part of the last paragraph ("The phenomenological models were based on the work of De Luca...") to the next section to ease the reading. This part is relative to the Phenomenological models only.*

**Response: **We have moved the paragraph to the phenomenological models’ section as suggested.

**

**Change in the manuscript:**

*“ The phenomenological models were based on the work of De Luca 37 where an sEMG signal from a muscle activated at a fixed level can be treated as zero-mean white noise followed by a shaping filter (electrode properties); this model is widely accepted in the literature. 24, 38 – 41… The first step in this model is the generation of the zero-mean unit-variance white Gaussian and Laplacian noise …”*

- *Figure 1 presenting examples of the three types of generated signals show the biophysical signal of a smaller (20 times) than the phenomenological ones. While the absolute amplitude likely does not affect the detectors, this is slightly confusing. Is there any valid reason for this difference or can these three signals be equalised to the same amplitude*

**Response:**

 The amplitude of biophysical model is in mV since it is modelled using physics of the EMG generation and recording process. Whereas with the phenomenological model the EMG signal is generated as bandpass filtered white noise, and thus its amplitude is in arbitrary units. We have now addressed this issue by scaling the biophysical models to have similar amplitudes to that of the phenomenological models to avoid any confusion.

- *In Table 1, authors refer to 'Weights' for \alpha whereas it appears that there is only a single value for each detector. Please clarify.*

**Response:**

 The optimal parameter for each detector type is selected by doing search over a range of values for each parameter. The performance of a detector is tested with different weights in the range ***α *** ∈{1,2,3,4,5}.  However, only one value by detector is used for computing the threshold. “Weights” in Table 1 has been modified as “Weight”.

- *In Table 1, while RMS and Lidierth detectors do use a filter for the conditioning, those filters cut-off frequencies are not listed as parameters. Do authors intentionally chose fixed, non-optimised, frequencies for those (and if so, why)? or is this a simple oversight in the table?*

**Response:**

 The Lidierth detector utilizes rectification for signal conditioning and does not incorporate a low-pass filter. This oversight in the table has been rectified. On the other hand, the RMS detector, as per the original paper, employed a band-pass filter between 10 Hz and 250 Hz (fs/2) on recoded raw EMG data to obtain the EMG signal within the required frequency spectrum. However, in this analysis, simulated data was generated by band-pass filtering white noise between 10 and 450 Hz. Therefore, the band-pass filter for signal conditioning in the RMS detector was redundant. Hence, we implemented the detector without the band-pass filter and achieved similar results.

- *After Eq 12, authors should clearly introduce \alpha and refer to it as weight as this is the first time it is properly introduced. I would also recommend to explicit it earlier on, as the Weight (weights?) are introduced in Table 1 earlier and earlier in the text but without explanation making it hard to follow.*

**Response:**

 Thank you, we have followed this suggestion as follows:

**Change in the manuscript:**

 The threshold *h* is adaptive and is calculated for each trial by adding α times the standard deviation of first 3 seconds data (Figure 3) to the mean. α is termed as *weight* for threshold in this paper. 

**Methods: On the cost definition:**

- *According to Eq. 16, f(deltat) is 0 (ideal) when the detection happens before the actual onset: \deltat<0ms and so is effectively a False positive. While I understand that this cannot be avoided with the chosen structure of the cost: this suggests that the within the overall cost, the latency and the false negative rates are more redundant whereas the false negative rate is quite independent of the other two.*

**Response:**

 The response is with the understanding that the reviewer's comment is as follows: “This suggests that the within the overall cost, the latency and the false negative rates are more redundant whereas the false positive rate is quite independent of the other two.”

 We performed a correlation analysis between false negative rate and the latency, which showed that:

- Moderate positive correlation between false negative rate and latency with 0 dB SNR signal of Gaussian and biophysical model (Figure 1).
- No correlation between FNR and latency with -3 dB SNR across all three models (Figure 2).

Therefore, the three factors are independent at lower SNR and there is a need to account for them separately in order to evaluate the performance of the detector. Scatter plots of FNR vs. latency at both 0dB and -3dB for one of the detector Hodges are presented in Figures 1 and 2, respectively. Similar results are observed for all other detectors.

*Fig.1. Correlation plot of false negative rate vs. latency of Hodges detector tested on the three signals model (from left: Gaussian, Laplacian and Biophysical) with 0dB SNR.*

*Fig.2: Correlation plot of false negative rate v.s latency of Hodges detector tested on the three signal model (from left: Gaussian, Laplacian and Biophysical) with -3 dB SNR.*

The figures can be found here

- *More generally, as the authors show that most detectors already behave quite poorly for such low SNR sEMG, the chosen cost bounds, and especially the 20% seems quite high for actual use. Given the application in mind where the objective is to detect one true onset as quickly as possible (and 50ms appears reasonable for a robotics assistance), I wonder if a cost accounting for a more strict false negative rate and more tolerant (if accounted at all) for false positive would make more sense. Indeed, only the earliest positive detection after onset can be considered useful for an application using this signal as a trigger. Wouldn't it make sense to also train and evaluate the detectors on a cost based solely on a very strict false positive rate (5%?) and not included FN in the cost (given that only the earliest detection after true onset matters)? Regarding the FP and FN rates, those are also calculated per trial, meaning that 20% (or even 5%) can mean that each trial has at least several FP before onset and not that 20% (or 5%) of the trials do have a FP. This is this latest figure that is of direct interest for the application. Authors should clearly discuss this point on how these results translate to real scenarios and provide results showing in what percentage of test trials FP detection happens before onset.*

**Response:**

 Firstly, we have modified the term EMG-triggered robot-assisted therapy  to EMG-driven robot-assisted therapy to emphasise that we are interested in implementing a therapy modality where the robot-assisted movement is contingent on the presence or absence of sEMG. Continued assistance required the continued presence of sEMG. This has been modified in the manuscript. We have also changed “sEMG-triggered” to “sEMG-driven”.

**Original text**:

 In sEMG-triggered robot-assisted therapy, robotic assistance is triggered whenever sEMG is detected in real-time in the target muscle during the move phase of a trial.

**Change in the manuscript:**

 In sEMG-driven robot-assisted therapy, the robot remains inactive during the rest phase, while in the move phase the robot-assisted movement is contingent upon the presence or absence of sEMG at any given time, thus continued sEMG is required to continuously receive robotic assistance.

**Response:**

 Making robot-assistance contingent on continued presence of sEMG is important to keep the subject actively involved during the entire duration of a task, and thus avoid slacking. Such a modality requires both the rejection of false positives, quick detection of sEMG onset, and the continued detection of sEMG when it is present (i.e. low false negatives). Low false negative rates may be essential for the user’s motivation and sense of agency.

 For higher SNR signals, the presence of the false negative rate may not be important in the cost function, but it is crucial for low SNR signals (0dB or -3dB) including the false negative rate.

 Also, the sample entropy, SSA and CWT detectors that performed worse across all three models had acceptable false positive rate and latency, but the false negative rate alone was more than 20% which contributed to higher cost. These detectors will also be considered suitable for application in robot assisted therapy if the false negative rate is not included in the cost. This suggest that it is important to optimised for false negative rate as well since we are looking at low SNR EMG signals.

 We have now included a short paragraph explaining why the three terms are included in the cost function:

**Original text**:

 The performance of a detector must consider the accuracy (false positive and false negative rate) and the detection latency.

**Change in the manuscript:**

 An optimal EMG detector designed for use in EMG-driven robot-assisted therapy should possess the capability to quickly identify the onset of sEMG, efficiently eliminate false positives, and consistently detect sEMG when it is present (lower false negatives). Such a detector is essential for maintaining user’s motivation and sense of agency.

**Response:**

 We agree that the 20% false positive rate might be too high. But since we are looking at very low SNR signal a strict FPR of 5% might decide all the detectors as not suitable for application in robot-assisted therapy. Also optimising for such low FPR could lead to parameter choices that compromise the true positive rate and latency since we are working with low SNR signals. Therefore, we have decided to use a more sensitive detector.

- We do not think that choosing a sensitive detector is a problem because the output of the detector will not be used to directly drive robot-assistance. Some form of low-pass filtering, or time-based filtering will be employed to smooth out jittery outputs from the detector before activating robotic-assistance. Such filtering operations on the detector output is commonly employed (e.g. Ramos-Murguialday et al., 2013). This can help filter out most short-duration false-positive pulses from activating robotic assistance. This filtering operation will increase the latency, which can impact the sense of agency and the perception of the user about the system’s responsiveness. Previous work indicates that a 200-300ms delay is well tolerated when reporting agency. Starting with a sensitive detector, we can experiment with different levels of filtering of the output with feedback from patients to yield an optimal detector. Individualising the detector and the filtering block is likely to favour patients to feel in control of their own movements.

We have now included this in the Discussion to explain the 20% cut-off choice for determining a good detector.

**Change in the manuscript:**

 In this analysis, a cost of 0.2 was selected for the application in sEMG-driven robot-assisted therap, corresponding to a 50 ms latency, 20% FPR or 20% FNR.  Permitting up to a 20% false positive rate might be too high, leading to the selection of a highly sensitive detector as the best choice. However, we  believe that choosing a sensitive detector  is not a problem, because the raw output of this detector is unlikely to be used directly to drive the robot-assistance. Some form of low-pass or time-based filtering (like the one employed by Ramos-Murguialday et al. ^1^) will be employed to filter out show false positives/negative pulses before using it to drive robotic assistance. This filtering operation reduce the FPR and FNR at the expense of introducing an additional latency; a delay of 200-300ms are well tolerated when reporting for sense of agency ^2^. The choice of amount of filtering of the chose detector’s output will need to be done through feedback from patients/users of the system.

 A 20% false positive rate does not imply that 20% of the trials have a false positive rate. Instead, we assessed the consistency of the detectors by determining the number of test trials with a cost less than 0.2. A suitable detector is identified as one that has 80% of test trials with a cost less than 0.2, as indicated by the grey boxes in Table 3. These highlighted detectors exhibit more than 80% of test trials with at most a 20% false positive rate. This means that every trial could have false positives, and in our analysis the best detectors are the ones that consistently show less than 20% false positives in each test trial.

**Discussion:**

- *It could be interesting to discuss the possibility that the signal processing (detectors) might not be necessarily implemented at the same frequency than the one the sEMG data is collected. Indeed, it is common in existing systems to have an EMG acquisition faster than the robotic controller (typically a >2kHz EMG sampling for a 1kHz controller). Do authors have clues on how that might differently affect the different detectors?*

**Response:**

 Although the EMG acquisition happens at faster rate when compared to the robotic controller **, **the performance of the different detectors is likely to be not affected by the sampling rate. Consider that the EMG detectors are implemented at the same frequency as the EMG acquisition system (1 kHz) but the robotic controller is running at lower sampling frequency of 100 Hz. Then the output of the EMG detector is to be down sampled to match the frequency of the robotic controller which might lead to increase in latency by few milliseconds (10 ms) which might not affect the overall behaviour of the EMG-triggered robot assistance system.

- *While the use of simulated (generated) EMG signals to evaluate the performance of the detector is appropriate and allows for robust and repeatable outcomes, it would be an interesting addition to also perform a short evaluation on actual low signal-to-noise EMG datasets.*

**Response:**

 We believe that evaluating the performance of the detector with real EMG datasets is outside the scope of this paper, in part as the same analysis approach cannot be applied with real data since we do not have full control over the amplitude and timing of the real EMG data, and we might not always have the ground truth. However, we are investigating if the outcomes of this study are supported by real data from patients from a previously concluded study.

- *On that point of potential difference with actual EMG signals, authors rightly point that they here test the optimal behaviour of the detectors, i.e. they would likely all perform worst on actual signals, especially when muscle activation is not constant over a long period. Can authors infer and discuss some difference in performance between the detectors for short activation (typically flickers that can be encountered in rehabilitation applications)? Some detectors are based on relatively long windows of 150ms, and while authors explain in the discussion that the best detectors have a latency of <50ms and so likely suitable for short activation bursts, how this would affect the detectors with longer window? Could they be likely be optimised differently for such signals?*

**Response:**

 From Table 3, we can see that the detectors that have more than 80% validation trials with cost consistently lower than 0.2 (latency < 50ms) have a window size of less than or equal to 100 ms as the optimised parameter (Table 2). AGLR-G detector under biophysical model (0dB and -3dB) and Laplacian model (-3dB) with window size of 150 ms do not fall under the detectors with acceptable cost. This shows that detectors with a larger window size are less likely suitable for short activation bursts. However, on an average across all three models AGLR-G detector had reasonable percentage of validation trials with cost less than 0.2, thus we have mentioned in the Discussion that it is also one of the detectors that is likely to pick up short bursts.

 We conducted an analysis on EMG signals with short pulses of 500 ms duration. The results indicated that optimised parameters differed for the step EMG and pulse EMG. We also included the offset time in evaluating the performance of the detector. When optimising for offset time, higher weights for the threshold and a greater cut-off frequency for the low-pass filter (smaller window sizes) were selected to minimise the offset time latency. Hence, the detectors that were optimised with step EMG with relatively longer window size may exhibit suboptimal performance on EMG, with intermittent muscle activity as they might miss to detect the short activation burst.

**Minor/typos:**

-  
*"Devasahayam S43"  => "Devasahayam43"*
- * In Table 2, some window sizes are in (ms) some in (s)*

**Response:**

 These have been corrected in the manuscript.
